# About making a CHO production cell line “research-friendly” by genetic engineering

**DOI:** 10.1186/1753-6561-5-S8-P132

**Published:** 2011-11-22

**Authors:** Bernd Voedisch, Agnès Patoux, Jildou Sterkenburgh, Mirjam Buchs, Emily Barry, Cyril Allard, Sabine Geisse

**Affiliations:** 1 Novartis Pharma AG, Basel, Switzerland; 2 University of Cambridge, Cambridge, United Kingdom; 3 Cardiff University, Cardiff, United Kingdom

## 

The use of Chinese Hamster Ovary (CHO) cells for transient gene expression is gaining importance steadily, as it has been shown that both quality and quantity of proteins derived from CHO cells differs from and can even be superior to material derived from Human Embryonic Kidney (HEK293) cells [1-4]. In order to augment yields HEK cell lines have been genetically modified, by e.g. stable integration of an expression cassette coding for the Epstein-Barr Virus Nuclear Antigen 1 (EBNA1) in conjunction with expression plasmid vectors containing the EBNA1 interaction site oriP. Here we describe the generation of a CHO cell line stably expressing the EBNA1 gene to enhance yields after large scale transient transfection with polyethylenimine (PEI).

An expression plasmid featuring the EBNA1 gene was transfected into an in-house available CHO wild type cell line by nucleofection and several stable pools were established by antibiotic selection. In a first approach, 133 clones were then isolated by limiting dilution cloning and subsequently expanded for further analysis. The approach aimed at identifying clones showing both the presence of EBNA1 protein and enhanced yields after PEI-mediated transient expression of a reporter protein at the same time.

Cell lysates or nuclear and cytoplasmic protein extracts from these clones were tested for presence of EBNA1 protein by Western Blot. An in-licensed cell line, CHO EBNALT85 (Icosagen AS, Tartu, Estonia) expressing the full length EBNA1 gene, and the HEK293-6E cell line (from the group of Y. Durocher, NRC, Canada) expressing a truncated EBNA1 gene served as positive controls. A number of commercially available anti-EBNA1 antibodies were tested in Western blotting, but most antibodies failed to detect the EBNA1 protein produced by these positive control cells. Only one antibody (Antibody 1EB12, Santa Cruz Biotechnology) was identified that detected the EBNA1 proteins in both positive control cell lines.

However, by applying this antibody on the 133 generated clones it became obvious that in most of them the EBNA1 protein appeared to be largely degraded.

The functionality of the EBNA1 protein in the cells was probed by PEI-mediated transient transfection of expression plasmids encoding the gene for the reporter protein Secreted Alkaline Phosphatase (SeAP). SeAP expression levels were compared with respect to presence or absence of the EBNA1 interaction sequence oriP on the SeAP expression plasmid. Only one clone showed a twofold enhanced production level of the reporter protein compared to the non-engineered parental CHO cell line. However, in this specific clone no EBNA1 protein could be detected by Western Blot, and the enhanced expression level was independent from the presence or absence of the oriP sequence on the SeAP expression plasmid. We therefore conclude that this clone does not possess a functional EBNA1 protein and an EBNA1/oriP based enhancement of productivity. The mechanism underlying the enhanced yield of the reporter protein in this particular clone remains currently elusive.

In an alternative approach flow cytometric cell sorting was applied to generate CHO clones producing a functional EBNA1 protein. Using the EBNA1 positive CHO cell line CHO EBNALT85 it could be shown that expressing eGFP from a transiently transfected expression plasmid with oriP sequence resulted in a 30- to 40-fold increase of highly fluorescent cells in comparison to an expression plasmid lacking the oriP sequence.

Thus, the already generated CHO pools stably transfected with the EBNA1 gene were transiently supertransfected with an eGFP expression plasmid containing the oriP sequence. Highly fluorescent cells were collected by cell sorting and expanded. Analysis by Western Blot revealed that this sub-pool was enriched for cells exhibiting the presence of a full length EBNA1 protein besides a major breakdown product. Transient transfection of this sub-pool with SeAP expression vectors led to the conclusion that the pool contained cells with a functional EBNA1 protein capable of enhancing SeAP yields in dependence on the presence of the oriP sequence on the SeAP expression plasmid. Clones were again generated from this sub-pool by limiting dilution cloning and characterized in analogy to the first approach. 9 out of 12 clones that were analyzed further showed increased levels of reporter protein production in dependence on the presence of the oriP element on the expression plasmid. The series of clones exhibiting highest expression levels will now be further evaluated by expression of other protein candidates.
